# Stemarane Diterpenes and Diterpenoids †

**DOI:** 10.3390/ijms20112627

**Published:** 2019-05-28

**Authors:** Francesca Leonelli, Alessio Valletta, Luisa Maria Migneco, Rinaldo Marini Bettolo

**Affiliations:** 1 Dipartimento di Biologia Ambientale, Università degli Studi La Sapienza, Piazzale Aldo Moro 5, I-00185 Roma, Italy; 2 Dipartimento di Chimica, Università degli Studi La Sapienza, Piazzale Aldo Moro 5, I-00185 Roma, Italy

**Keywords:** stemarane, diterpenes, diterpenoids, isolation, structure, biogenesis, biosynthesis, synthesis, enantiosynthesis

## Abstract

In this article the scientific activity carried out on stemarane diterpenes and diterpenoids, isolated over the world from various natural sources, was reviewed. The structure elucidation of stemarane diterpenes and diterpenoids was reported, in addition to their biogenesis and biosynthesis. Stemarane diterpenes and diterpenoids biotransformations and biological activity was also taken into account. Finally the work leading to the synthesis and enantiosynthesis of stemarane diterpenes and diterpenoids was described.

## 1. Introduction

**Figure 1:**
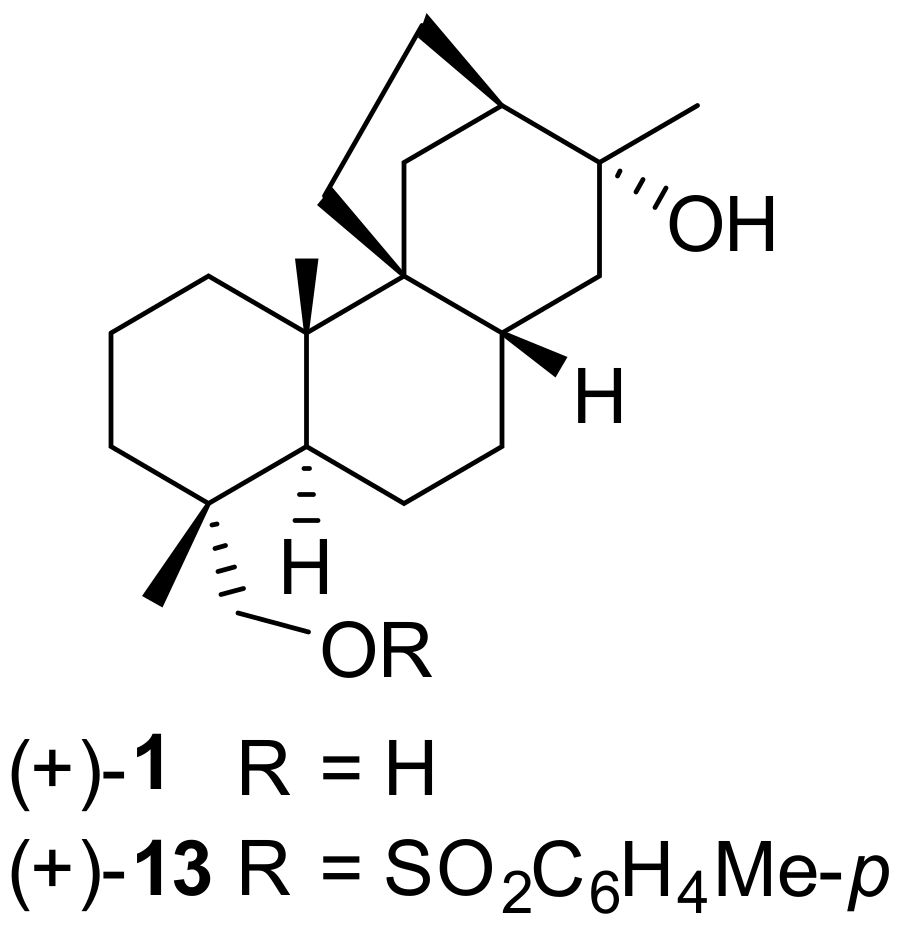
(+)-stemarin (**1**) and corresponding C(18) tosylate (**13**).

**Figure 2:**
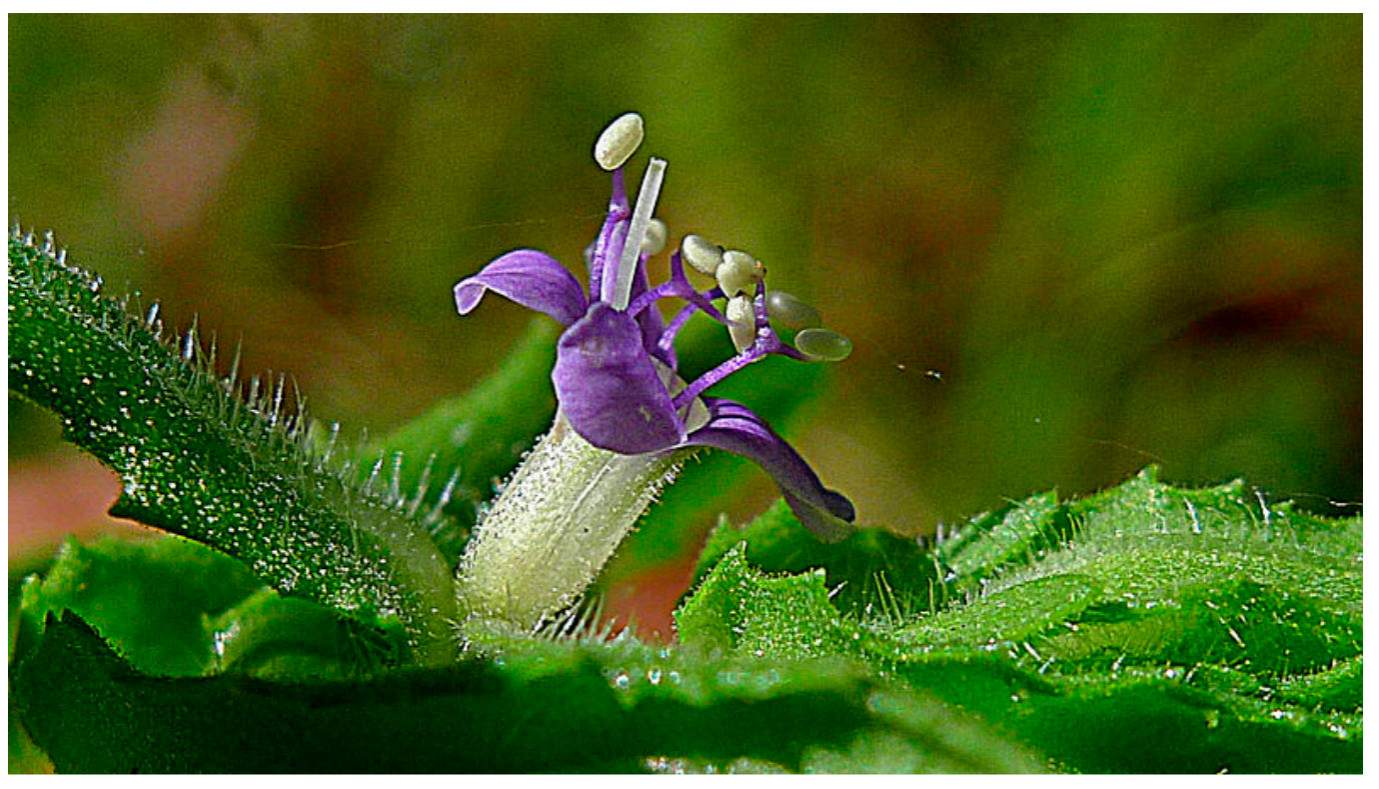
*Stemodia maritima* L. (*Scrophulariaceae*). (From: Alex Popovkin, Bahia, Brazil, available online: https://flickr.com/photos/12589168@N00/15015148887.

Stemarane diterpene and diterpenoids are tetracyclic compounds characterized by a unique hydrocarbon skeleton. They were named after (+)-stemarin **1** (Figure 1), isolated in 1975 by Manchand and Blount [1] at Hoffmann-La Roche Inc., Nutley, N.J. from *Stemodia Maritima* L. (*Scrophulariaceae*) (Figure 2), a rare West Indies littoral plant.

**Figure 3:**
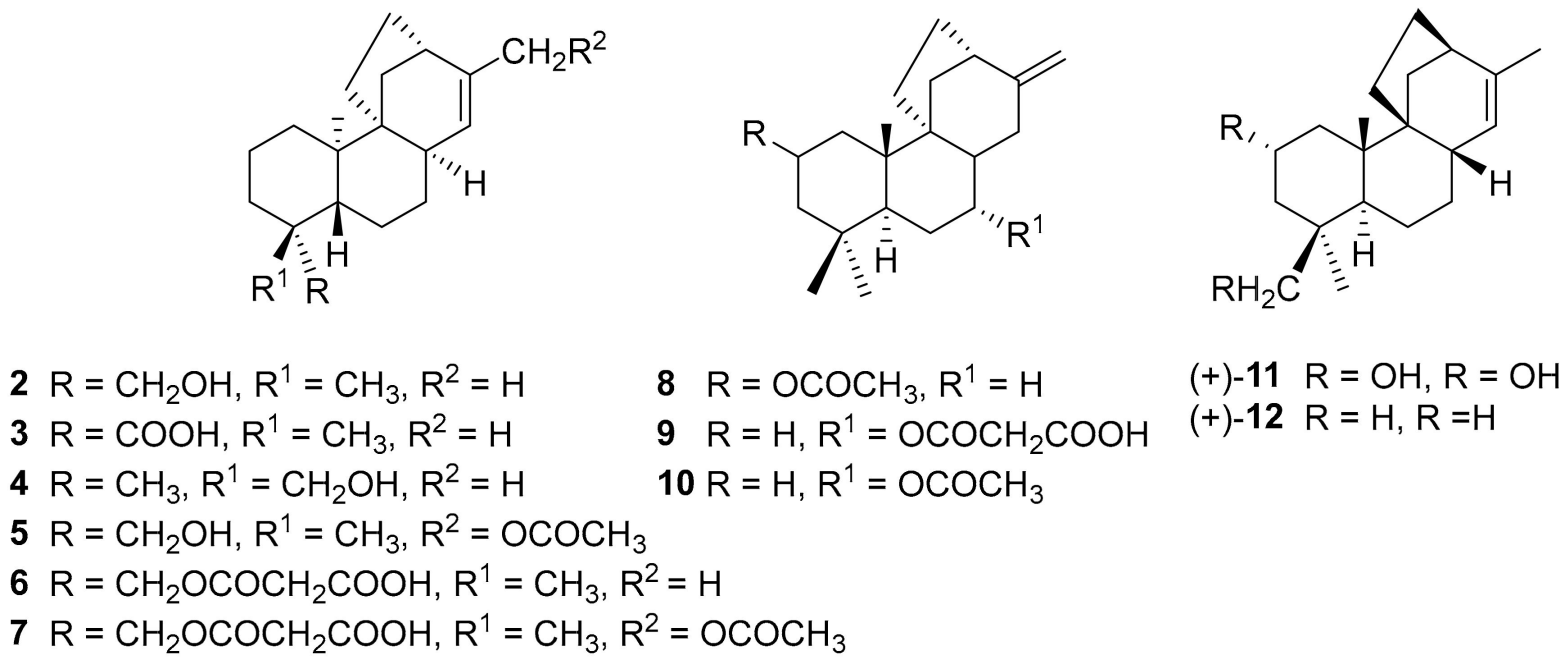
*Ent*-stemarane diterpenoids isolated from Chilean plants of *Calceolaria* genus (**2**–**10**) and (+)-oryzalexin S (**11**) and (+)-13-stemarene (**12**).

Some years later Garbarino and co-workers isolated—at Universidad Técnica Federico Santa Maria, Valparaiso—a number of stemarane diterpenes from Chilean plants of the *Calceolaria* genus (Figure 3), whose absolute configuration was opposite to that of (+)-stemarin (**1**). Thus, from the aerial parts of *Calceolaria lepida* Phil., a plant that grows in the coastal hills of central Chile, the Valparaiso group isolated *ent*-stemar-13(14)-en-19-ol **2** and *ent*-stemar-13(14)-en-19-oic acid **3** [2]. The former was also isolated, along with and (+)-*ent*-stemar-13(14)-en-18-ol **4**, from *Calceolaria latifolia* Benth. [3], a species growing on hills of north and central Chile.

The Valparaiso group also reported the isolation and structure elucidation from *Calceolaria kingii* Phil., which grows in north and central Chile, of *ent*-stemara-13(14)-en-17-acetoxy-18-ol **5** [4]; from *Calceolaria polifolia* Hook, a species which grows on the coastal hills of central Chile, of 19-malonyloxy-*ent*-stemar-13(14)-ene **6**, 17-acetoxy-19-malonyloxy-*ent*-stemar-13(14)-ene 7, and already known *ent*-stemar-13(14)-en-19-ol **2** (*vide supra*) [5]; from the aerial parts of *Calceolaria dentata* Ruiz & Pav., a perennial plant which grows in the middle-South of Chile, of 2β-acetoxy-13-methylene-stemarane **8** and 7-malonyloxy-13-methylene-stemarane **9** [6] (In references [6,7], though a stemarane structure was attributed to compounds **8**, **9** and **10**, the stereochemistry of H-C(8) was not specified in the formulae, and the C(9)-C(11) two carbon bridge was drawn with stereochemistry opposite to the CH_3_-C(10) as in stemarane diterpenes and diterpenoids.); from *Calceolaria glabrata* Phil. var. *glabrata*, a shrub common in the southern part of Chile, of 13-methylene-7-acetoxy-stemarane **10** [7] and finally from *Calceolaria paralia* Hook of *ent*-stemar-13(14)-en-19-oic acid **3** [8].

**Figure 4:**
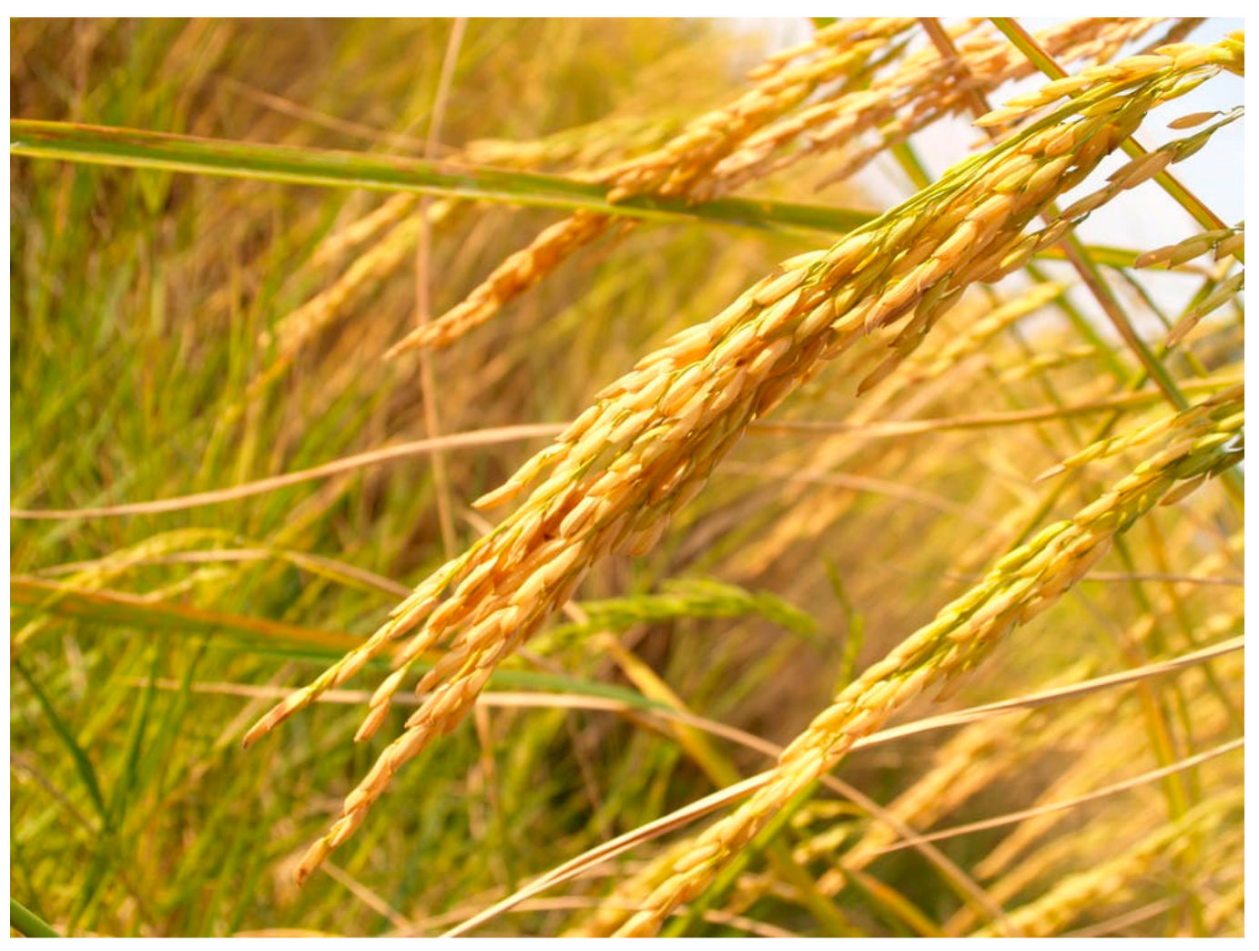
*Oryza sativa*. (From: https://www.pexels.com/photo/close-up-photo-of-rice-grains-during-daytime-162992/).

In the following years Tamogami and co-workers at Central Research Laboratories, Idemitsu Kosan Co., Ltd., Chiba and at Ibaraki University, Ibara, isolated from *Pyricularia oryzae* Cav. attacked *Oryza sativa* L. (*Poaceae*) (Figure 4) (+)-oryzalexin S [9] **11**, a stemarane diterpenoid which displays phytoalexin properties. The production by the plant of (+)-**11** is also induced by UV irradiation [9]. The production of diterpenoid phytoalexins after induction by UV irradiation was also studied in five rice genotypes of different susceptibility to the rice blast fungus *Pyricularia oryzae* at Reading University by Harborne and his group [10].

Finally in 2001 Oikawa, Sassa, and co-workers, at Hokkaido and Yamagata Universities respectively, reported the isolation of (+)-13-stemarene **12** [11] from the phytopathogenic fungus *Phoma betae*. 

## 2. Structure

**Figure 5:**
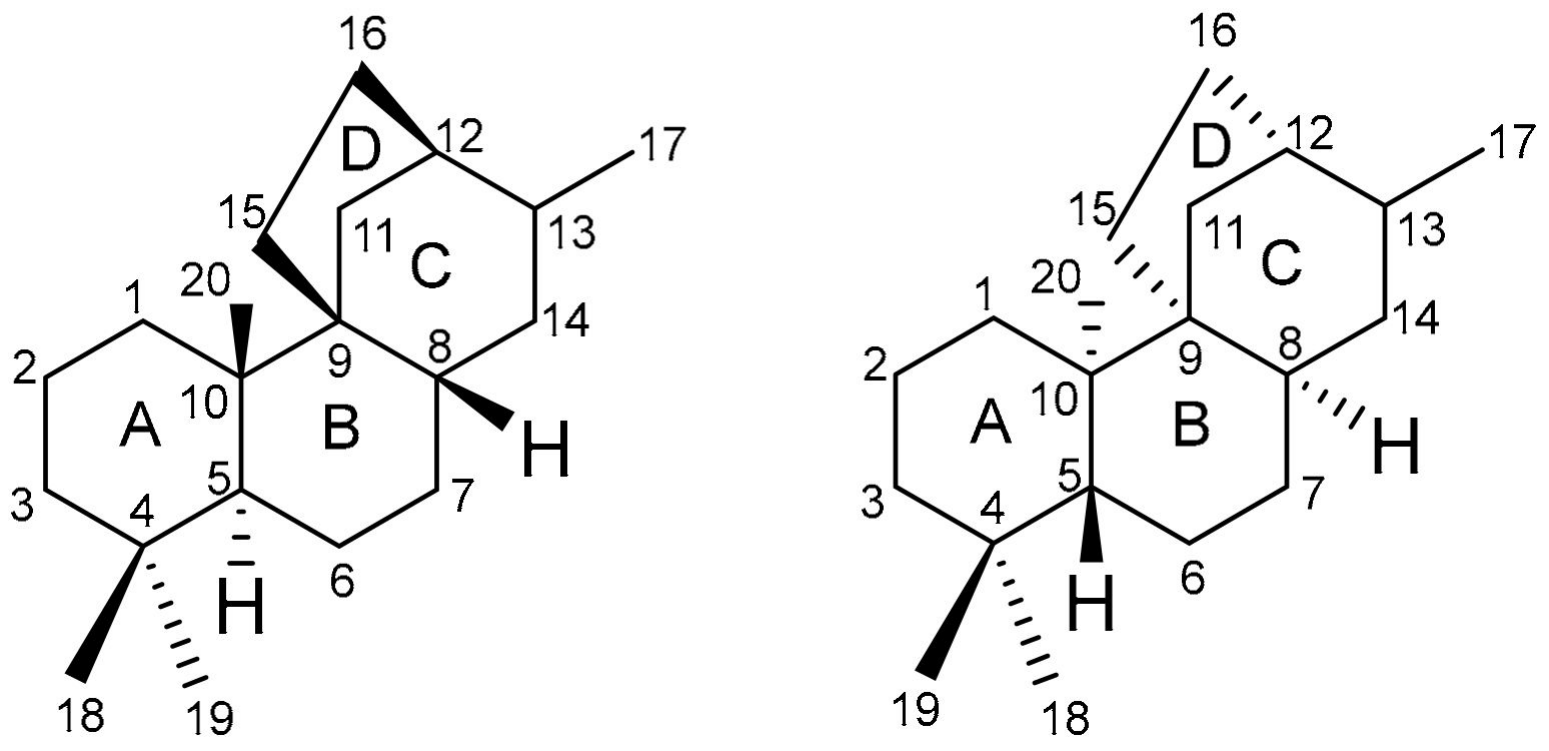
Stemarane (left) and *ent*-stemarane (right) diterpenes carbon skeleta.

The stemarane diterpenes carbon skeleton is reported in Figure 5, and its main structural features are reported below: the bicyclic system C/D is constituted by a bicyclo[3.2.1]octane fused to the bicyclic A/B system in a different fashion with respect to other tetracyclic diterpenes possessing the bicyclo[3.2.1]octane system; two contiguous quaternary carbon atoms, the C(9) and C(10), are present, the former being a spirocyclic atom; oxygenated functions can be present at C(2), C(7), C(13), C(17), C(18), and C(19); the A/B ring junction is *trans*; the H-C(8) is *syn* to the CH_3_-C(10), and to the two carbon bridge connecting C-(9) and C-(12) (In this review only compounds with such features are reported.).

The structure and absolute stereochemistry of (+)-stemarin **1** was assigned by Manchand and Blount after a chemical study and an X-ray crystallographic analysis on tosylate (+)-**13** [1].

The structure and relative configurations of stemarane diterpenoids **2**–**10** were assigned by Garbarino and co-workers by means of ^1^H- and ^13^C-NMR (Nuclear Magnetic Resonance), mass spectroscopy and by comparison with the spectral data of (+)-**1**. On the basis of biogenetic considerations, the *ent* absolute configuration was also assigned. No chemical correlations and/or X-ray structure determinations were ever made.

The structure proposed for (-)-**2** [2,3,5] was not confirmed by the synthesis (*vide infra*) from (+)-podocarpic acid of (+)-2-deoxyoryzalexin S **2**, reported by our group [12].

The structure and relative configuration of (+)-oryzalexin S **11** was established by a series of 2D-NMR experiments, which proved the relative configuration [13]. The structure and relative configuration of (+)-13-stemarene **12** was established by ^1^H-NMR [11]. The comparison with an authentic sample, obtained by our group from commercially available (+)-podocarpic acid, confirmed the proposed structure as well as its absolute configuration [14].

## 3. Biogenesis

**Scheme 1:**
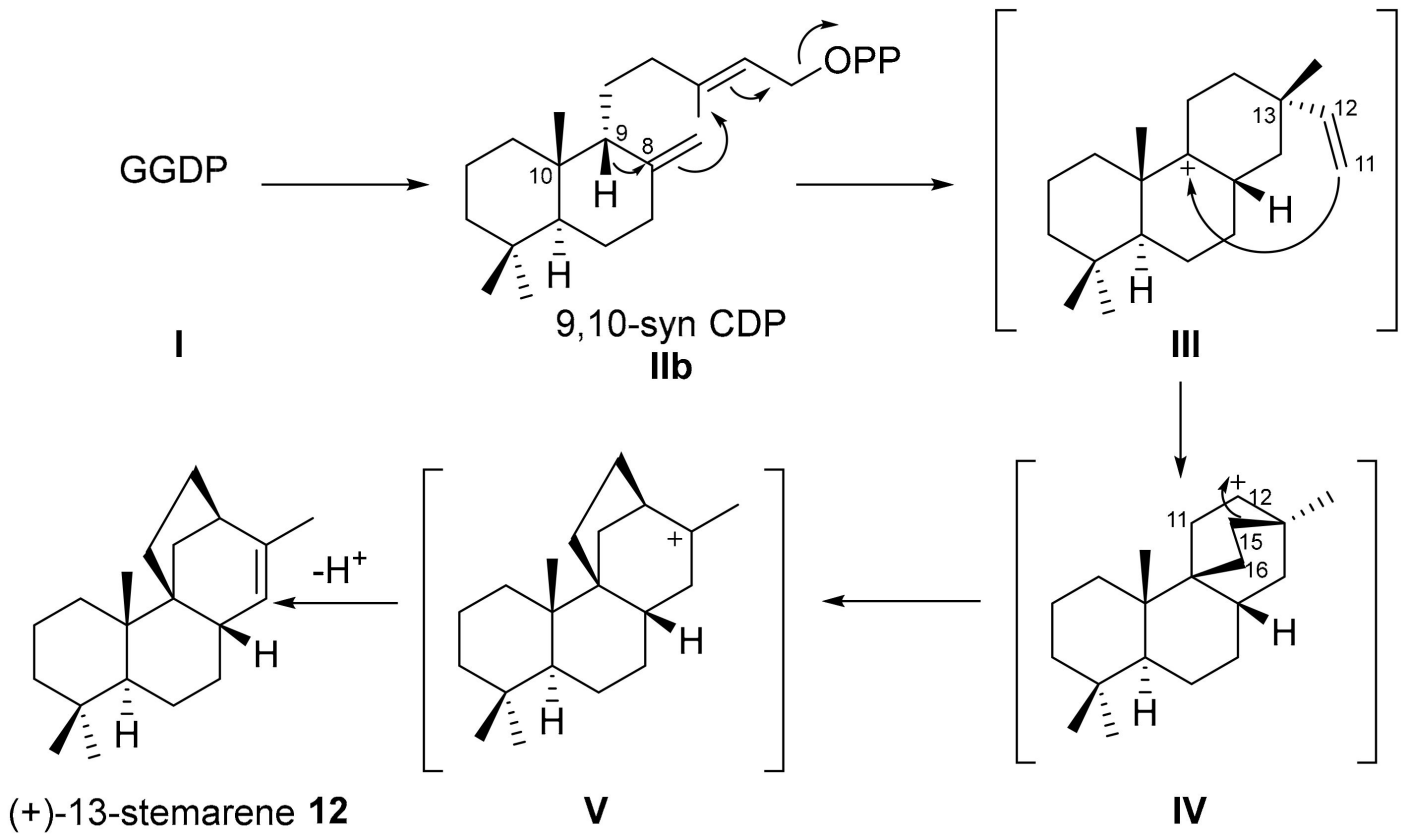
Biogenetic pathway to (+)-13-stemarene 12 as proposed by Tamogami and co-workers [13]. The black curved arrow represents the electrons’movement.

The biogenesis of stemarane diterpenes and diterpenoids, outlined in Scheme 1, was proposed by Tamogami and co-workers in 1993 [13]. The Authors proposed the biogenesis of this compound from GGPP (**I**) and compared it with the biogenesis of the other rice labdane-related diterpenoids. Since neither a proton nor a vinyl group is present at C(9) in (+)-oryzalexin S (**11**), the Authors assumed that its biogenesis begins with a hydride rearrangement from C(9) to C(8) by which **II** is converted into **III** in which the CH_3_-C(13) and the vinyl-C(13) group are α and β oriented, respectively. The attack to the C(9) carbocation by the vinyl group at C(13) gives then intermediate **IV**. A further rearrangement leads to intermediate **V** from which (+)-13-stemarene **12** is formed by the loss of H^-^. Oxidative processes at C(2) and C(19) give then (+)-oryzalexin S **11**.

## 4. Biosynthesis

**Scheme 2:**
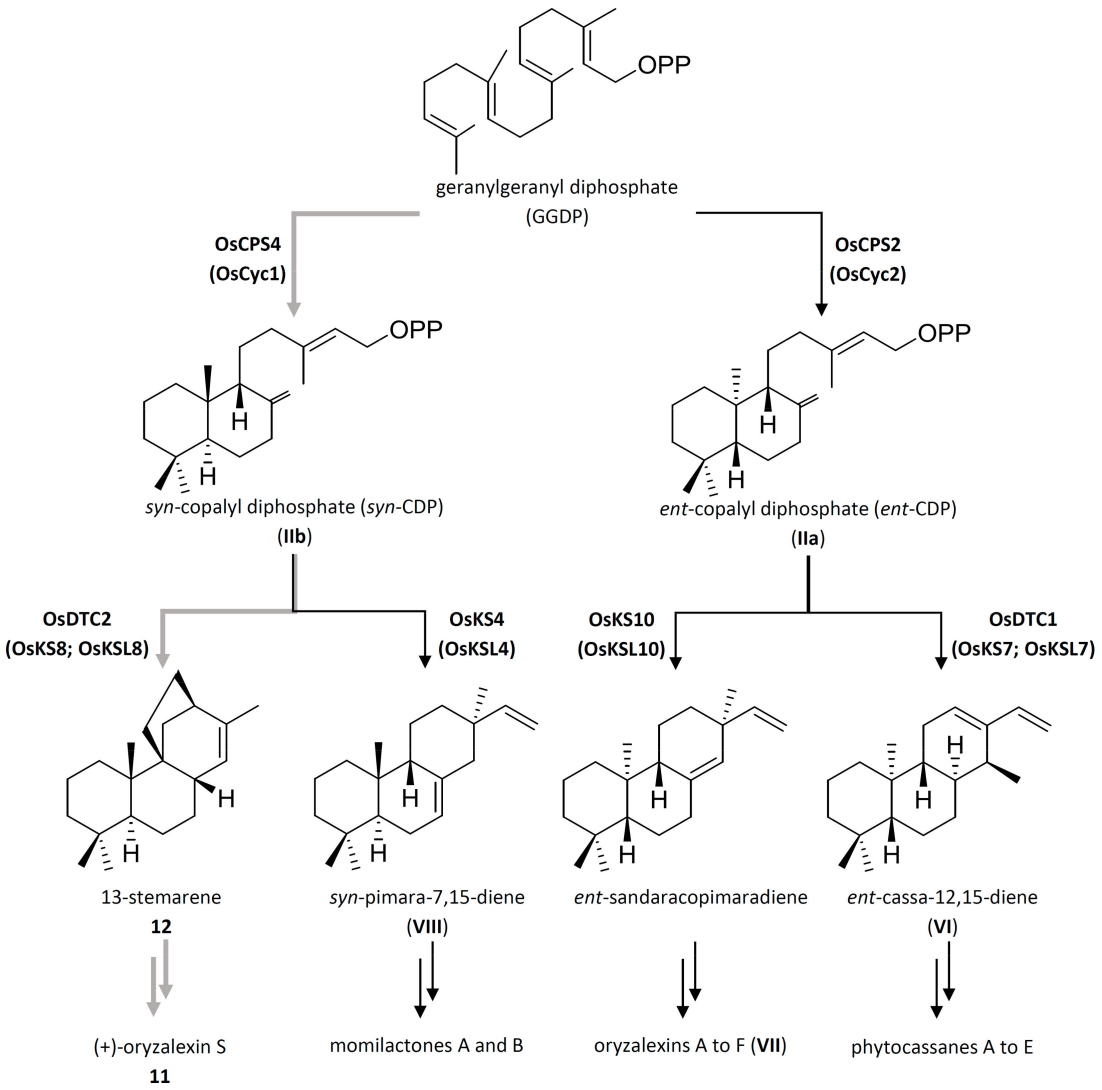
Proposed pathway for the biosynthesis of diterpenoid phytoalexins in rice. Grey arrows indicate the branch that leads to (+)-oryzalexin S **11**, while black arrows indicate the branches that leads to other labdane-related diterpenoids OsCPS2 (OsCyc2): *ent*-copalyl diphosphate synthase 2; OsCPS4 (OsCyc1) *syn*-copalyl diphosphate synthase; OsDTC1 (OsKSL7; OsKS7): *ent*-cassa-12,15-diene synthase; OsKS10 (OsKSL10): *ent*-sandaracopimaradiene synthase; OsKS4 (OsKSL4): *syn*-pimara-7,15-diene synthase; and OsDTC2 (OsKSL8; OsKS8) 13-stemarene synthase.

The few available studies on stemarane diterpene biosynthesis have been carried out on rice (*Oryza* spp.) plants and cell suspension cultures [15,16,17,18]. Rice diterpenes mainly belong to the class of labdane-related diterpenoids and most of them act as phytoalexins, i.e., molecules involved in plant defence whose biosynthesis is induced or enhanced by pathogen infection [19,20,21], treatment with signal molecules derived from pathogens (elicitors) [16], or exposure to UV radiation [9,16,22,23,24]. Gibberellins, a class of ubiquitous plant growth regulators, also belong to labdane-related diterpenoids [25]. On the basis of the structure of their hydrocarbon precursors, labdane-related diterpenoids are classified into four groups (Scheme 2): oryzalexins A to F [19,23,24], (–)-phytocassanes A to E [20,21,26,27], momilactones A and B [22,28], and (+)-oryzalexin S **11** [13].

Labdane-related diterpenoids are biosynthesized from (E,E,E)-geranylgeranyl diphosphate (The molecule is also referred in the bibliographic sources as geranylgeranyl pyrophosphate) (GGPP), the universal diterpene precursor, which is cyclized to *ent*-copalyl diphosphate (*ent*-CDP) or *syn*-copalyl diphosphate (*syn*-CDP) by the labdane-related diterpene cyclases ent-copalyl diphosphate synthase 2 (OsCPS2) and syn-copalyl diphosphate synthase (OsCPS4), respectively. These cyclized diphosphate compounds can then be further cyclized and/or rearranged by more typical class I terpene synthases, which initiate catalysis via ionization of the allylic pyrophosphate group [29]. *Ent*-CDP is converted to *ent*-cassa-12,15-diene by ent-cassa-12,15-diene synthase (OsDTC1) or to *ent*-sandaracopimaradiene by ent-sandaracopimaradiene synthase (OsKS10), leading to phytocassanes A to E or oryzalexins A to F. *Syn*-CDP is converted to *syn*-pimara-7,15-diene by syn-pimara-7,15-diene synthase (OsKS4) or to (+)-13-stemarene **12** by 13-stemarene synthase (OsDTC2), leading to momilactones A and B and (+)-oryzalexin S **11**.

In 1996, Mohan and co-workers from the University of Illinois reported the biosynthesis of cyclic diterpenes with enzyme extracts from rice cell suspension cultures to verify proposed pathways and intermediates in the production of momilactone and oryzalexin phytoalexins [15]. In the course of this work, the authors could confirm the role of 9,10-syn-copalyl diphosphate (syn-CDP) as a precursor of, *inter alia*, (+)-13-stemarene **12**, from which (+)-oryzalexin S **11** is presumed to be formed.

In 2004, Yamane and co-workers from the University of Tokyo reported that two species of mRNA encoding OsDTC1 and OsDTC2, two putative diterpene cyclases, were expressed in rice cell suspensions in response to chitosan-elicitation [30]. The same authors obtained OsDTC2 cDNA from chitin-elicited suspension-cultured rice cells [16]. They overexpressed OsDTC2 cDNA in *Escherichia coli* as a fusion protein with glutathione S-transferase and demonstrated that the recombinant protein function as stemar-13-ene synthase, the enzyme that catalyse in the conversion of *syn*-CDP into (+)-13-stemarene **12**, the putative precursor of (+)-oryzalexin S **11** (Scheme 2). They also observed (+)-13-stemarene **12** accumulation in both chitin-elicited suspension-cultured rice cells and in UV-irradiated rice leaves.

The carbocation reaction network for the formation of C(9)-ethano-bridged diterpenes, including stemarane diterpenes was described in 2002 by Oikawa and co-workers who integrated chemical and computational methods [31]. Finally, in 2018 Young and Tantillo from the University of California shed new light on the mechanisms of formation of the stemarene, stemodene, betaerdene, aphidicolene, and scopadulanol diterpenes from *syn*-CDP. The Authors demonstrated that the compounds of interest are interconnected by a complex network of reaction pathways, and that the interconnection of these paths leads to multiple routes for formation of each diterpene, which could lead to different origins for some carbon atoms in a given diterpenes under different conditions [32].

## 5. Biotransformations

**Figure 6:**
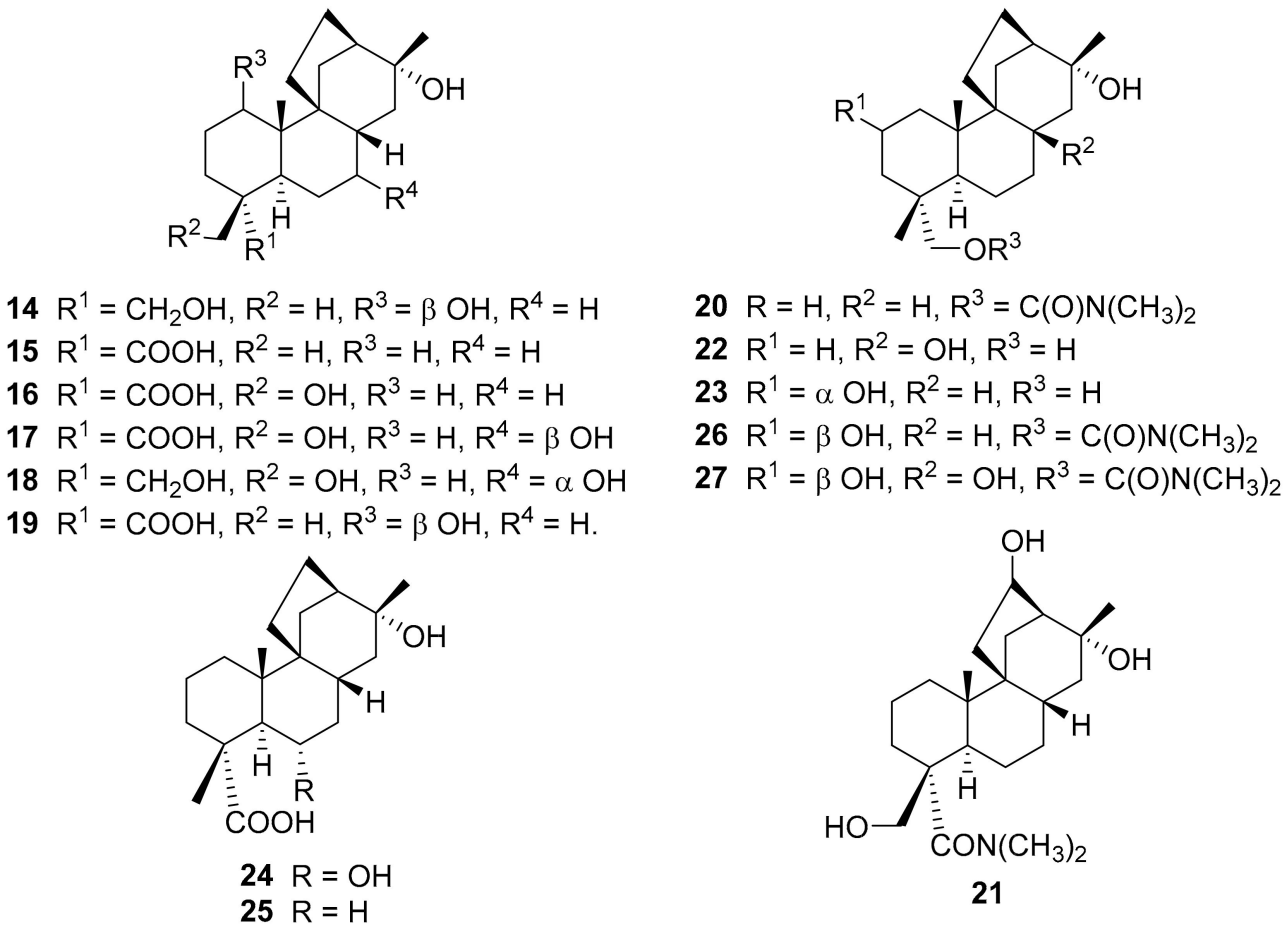
Metabolites obtained by the action of several fungi on (+)-stemarin (**1**) and derivatives.

Extensive work in the area of biotransformations was carried out by Reese and his group at the University of West Indies, Mona. The Reese group reported the biotransformation by the action of *Beauveria bassiana* ATCC 7159 of (+)-stemarin **1** into **14** and **15** [33] (Figure 6). The same group reported that, by the action of *Aspergillus niger* ATCC 9142, (+)-**1** is converted into four new metabolites (**16**, **17**, **18**, **19**) while the (+)-dimethylcarbamate **20** gives **21** [34].

Later Reese and co-workers reported, *inter alia*, the hydroxylation of (+)-**1** by *Mucor plumbeus* ATCC 4740 [35]. The incubation of (+)-**1** with the fungus produced two new metabolites (+)-**22** and (+)-**23** to which on the basis of HRMS (High resolution mass spectrometry) data ^13^C and 2D NMR the structures of (+)-8,13,19-trihydroxystemarane and (+)-2α,13,19-trihydroxystemarane were attributed, respectively. On the contrary, the dimethylcarbamate derivative (+)-**20** was not metabolised by the fungus. These biotransformations have been later reviewed [36]. In the following year, the Reese group described [37], *inter alia*, the bioconversion by *Cunnighamella echinulata* var. *elegans* of (+)-**1** into (+)-6α,13-dihydroxystemaran-19-oic acid **24** while (+)-**20** gave (+)-13-hydroxystemaran-19-oic acid **25** along with metabolites (+)-19-(*N*,*N*-Dimethylcarbamoxy)-2β,13-dihydroxystemarane **26** and (+)-19-(*N*,*N*-Dimethylcarbamoxy)-2β,8,13-trihydroxystemarane **27**. No bioconversions were obtained from the fermentation of (+)-**1** and (+)-**20** with *Phanerochaete chrysosporium*.

**Scheme 3:**
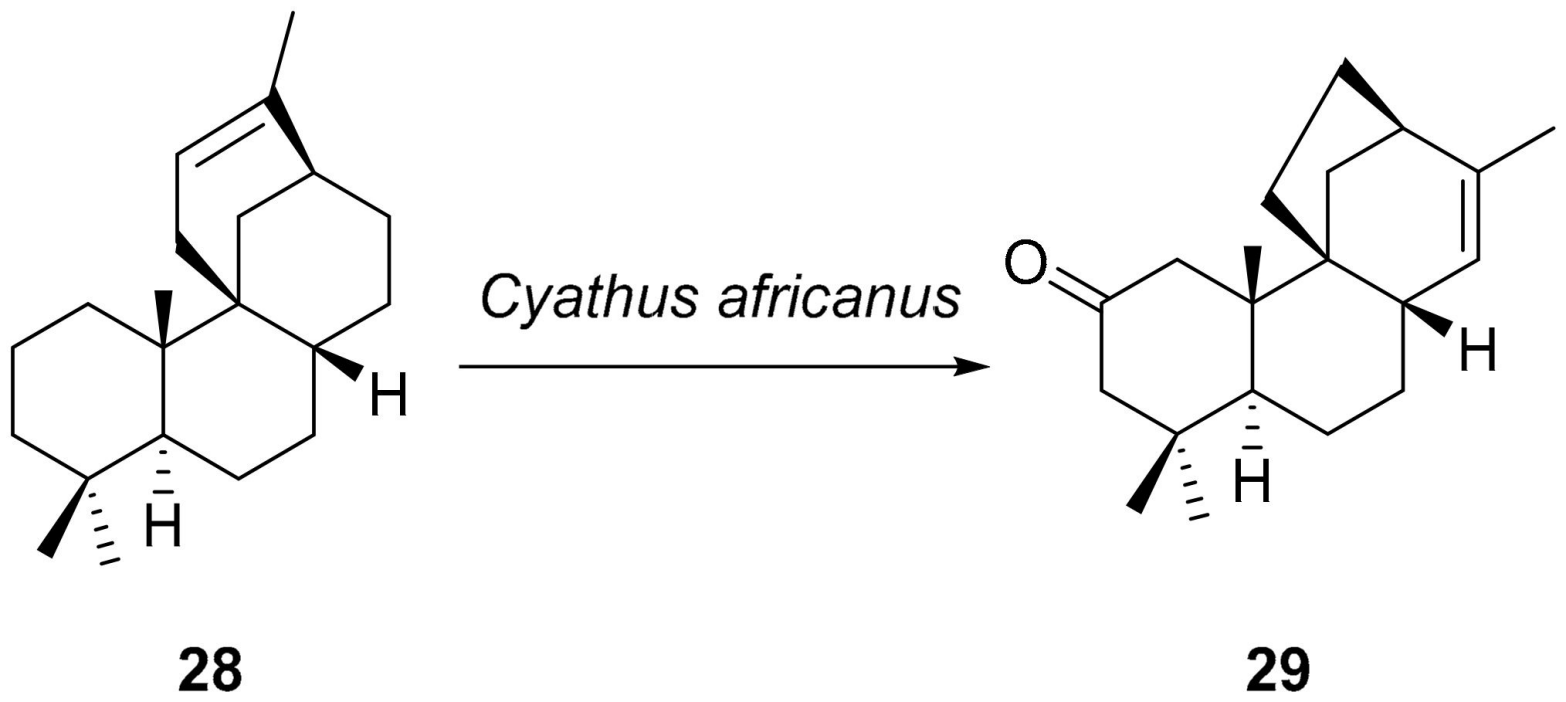
Conversion of 12-stemodene **28** into 2-oxo-13-stemarene **29** by the fungus *Cyathus africanus*.

Finally, a very interesting observation was made by the Reese group; 12-stemodene **28**, under the conditions the biotransformation by the fungus *Cyathus africanus* is carried out, not only is oxidized at C(2) but the skeleton is rearranged to the stemarene one giving 2-oxo-13-stemarene **29** (Scheme 3) [38]. In this respect, it should be recalled that stemodane and stemarane diterpenoids were both isolated from *Stemodia maritima* L. [1,39,40].

## 6. Biological Activity

In the folk medicine of Dutch Antilles an infusion of leafy branches of sea lavander (*Lavandula*) and *Stemodia maritima* L., mixed with Epsom salts, is used by men against venereal diseases [41]. Plants of *Calceolaria* genus are used in Central and South America popular medicine as stomach tonics, bactericidal agents, and sweeteners [42]. Nevertheless, to our best knowledge, the biological activity of pure isolated stemarane diterpenes was not investigated with the exception (+)-oryzalexin S 11 which, as stated above, was found to possess phytoalexin activity [9].

Essential oils obtained by a Brazilian research group, leaded by Arriaga at Universidade Federal do Ceará, Fortaleza, from *Stemodia maritima* L. leaves and stems, collected in the state of Ceará, showed larvicidal properties against the larvae of the mosquito *Aedes aegypti*, responsible for the transmission of yellow fever in Central and South America and in west Africa and a vector of dengue hemorrhagic fever [43]. Nevertheless, the major components found in the leaf oil were β-caryophyllene and 14-hydroxy-9-*epi*-β-caryophyllene, while in the stem oil β-caryophyllene and caryophyllene oxide were the most abundant. This biological activity cannot therefore be attributed to stemarane diterpenes and diterpenoids.

Besides, the same research group evaluated the antioxidant and antibacterial activity of some *Stemodia maritima* L. isolated metabolites, but stemarane diterpenoids [44], and could also observe that *Stemodia maritima* L. extracts decrease inflammation, oxidative stress, and alveolar bone loss in an experimental periodontitis rat model [45].

It appears, therefore, that the biological activity of pure isolated stemarane diterpenes and diterpenoids, but (+)-oryzalexin S **11**, has not been evaluated yet. The biological activity ascertained so far appears due to *Stemodia maritima* L. metabolites is different from that of stemarane diterpenes and diterpenoids. Comparing the content of stemarane diterpenes and diterpenoids among *Stemodia maritima* L. plants, collected in different geographical areas and extracted with the same procedure, seems a due task.

## 7. Synthesis

The C/D ring mojety constituted by a bicyclo[3.2.1]octane system fused in a novel way to the ring B, the presence of various stereocenters, two of which are the adjacent quaternary carbons (C-9 and C-10) and the interesting biological activity of some terms of this class of compounds, make stemarane diterpenoids a worthy synthetic challenge.

Kelly and co-workers at the University of New Brunswick (St. John N.B., Canada) and our group, both belonging to the Wiesner [46] school, got engaged with the synthesis of these very interesting compounds by the approach had been developed by their Mentor for the construction of the C/D ring system of diterpene alkaloids [47,48,49,50,51,52,53].

The approach adopted at first for obtaining this class of diterpenoids was based on the following steps:

(a) Allene photoaddition to a suitably substituted α,β-unsaturated carbonyl intermediate [54,55,56,57,58,59,60];
(b) Elaboration of the resulting photoadduct into a 1-methyl-6-hydroxybicyclo[2.2.2]octan-2-one [61,62];
(c) Rearrangement of the 1-methyl-6-hydroxybicyclo[2.2.2]octane intermediate or suitable derivative into a bicyclo[3.2.1]octan-2-ene [63,64,65,66].

### 7.1. The (±)-Stemarin 1 Total Synthesis by Allene Photoaddition to a 9(11)-Podocarpen-12-one Intermediate

**Scheme 4:**
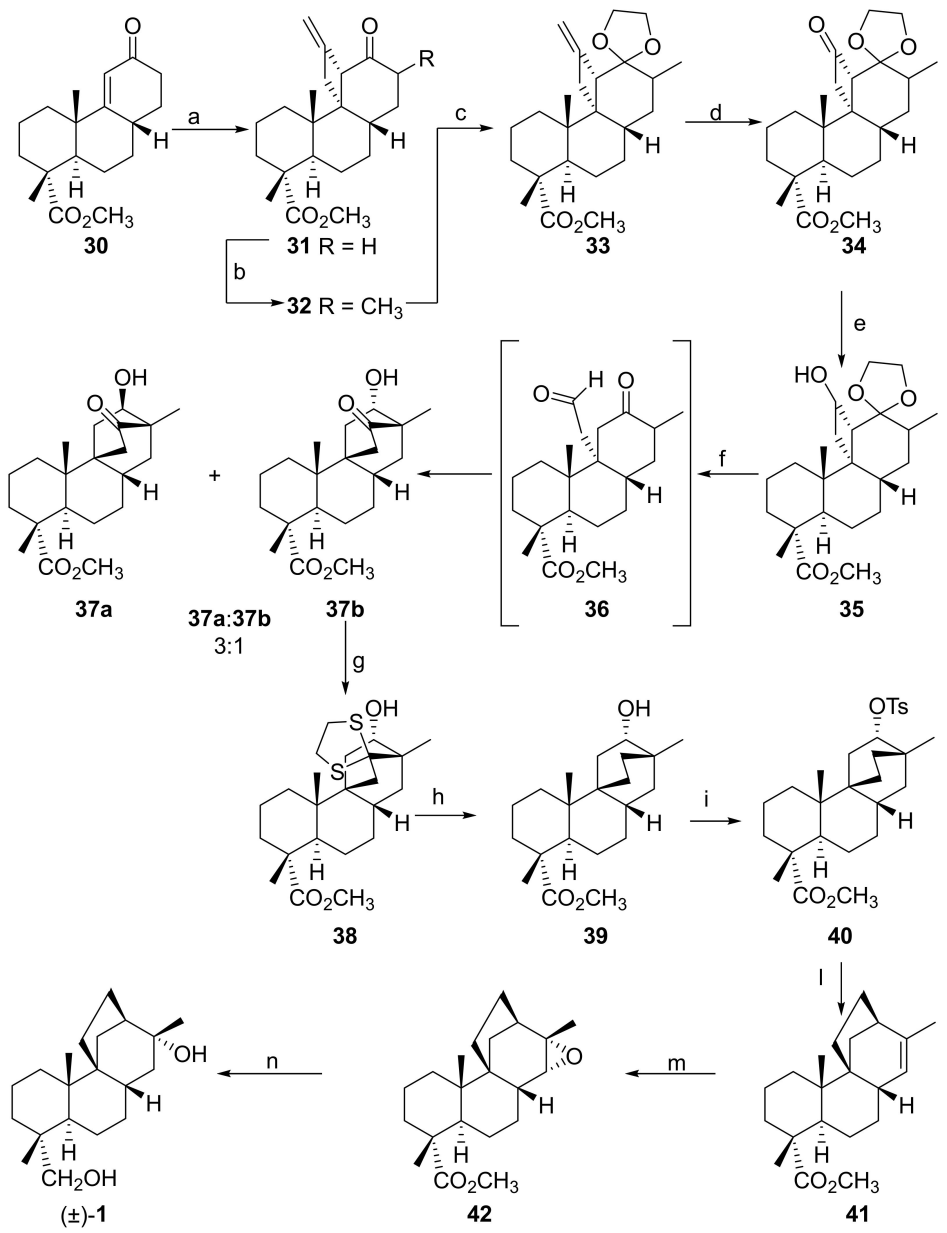
Schematic representation of (±)-stemarin **1** synthesis by Kelly and coworkers. Reagents and conditions: (**a**) allene, hν (Photochemical reaction), −78 °C; (**b**) THF (Tetrahydrofuran), −10 °C, lithium diisopropylamide, MeI; (**c**) (CH_2_OH)_2_, TsOH; (**d**) OsO_4_, NaIO_4_; (**e**) NaBH_4_; (**f**) HCl, THF; (**g**) (CH_2_SH)_2_, BF_3_·Et_2_O; (**h**) *Raney*-Ni, EtOH; (**i**) TsCl, Py; (**l**) NaCH_2_SOCH_3_, dimethyl sulfoxide; (**m**) meta-chloroperoxybenzoic acid; and (**n**) LiAlH_4_, Et_2_O.

The first synthesis of a stemarane diterpene was disclosed in 1980 by Kelly and co-workers [67]. The St. John N.B. group obtained (±)-stemarin **1** from known racemic tricyclic intermediate **30** [68] (Scheme 4). Tricyclic enone **30**, was submitted to allene photoaddition at −78 °C. The resulting photoadduct **31**, whose stereochemistry followed from its conversion into the final compound, was methylated at C(13) to give **32**. After protection of the carbonyl group (**33**), the exocyclic methylene was oxidatively cleaved to give **34**. The latter was than reduced to the masked ketol 35. Thus, under acidic conditions the ketal group was removed and the resulting ketol underwent a retroaldol-aldol reaction to give ketols **37a** and **37b** in an about 3:1 ratio. Only the aldol **37b** has the correct hydroxyl configuration for the subsequent rearrangement to the stemarin skeleton. The synthesis was therefore continued with **37b**. Deoxygenation led to alcohol **39** which was converted into the tosylate **40**. The latter upon rearrangement gave the olefinic ester **41**. The conversion of this compound into (±)-stemarin **1** was then accomplished by stereoselective epoxidation to give **42** and hydride reduction of the latter. The synthetic material was found to be identical to an authentic sample by comparing the IR (Infrared radiation) and NMR spectra and by the identical behavior on TLC (Thin-layer chromatography) in a variety of solvents.

### 7.2. Approaches for Diastereoselective Syntheses of (+)-13-Stemarene 12 and (+)-18-Deoxystemarin 2 and (±)-Stemarin 1 by Allene Photoaddition to 9(11)-Podocarpen-12-one and 8(9)-Podocarpen-14-one

While Kelly and his group were engaged in the synthesis of (±)-stemarin **1**, our group was involved in the synthesis of stemodane [69,70,71] diterpenoids and aphidicolin [69,72,73]. After the successful conclusion of this work, a diastereoselective route to the key 6-hydroxy-1-methylbicyclo[2.2.2]octane intermediate (missing in the Kelly approach) appeared to us a worthwhile synthetic challenge. (+)-13-stemarene **12** and (+)-18-deoxystemarin **2**, the simplest terms in the class were chosen as targets.

**Scheme 5:**
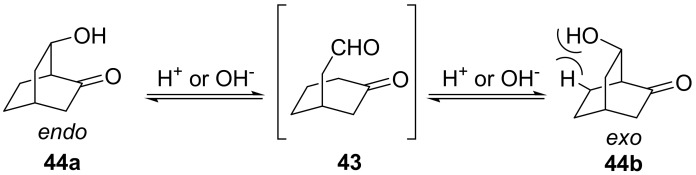
*Endo/exo* equilibration of 6-hydroxybicyclo[2.2.2]octan-2-ones under either basic or acid conditions.

As mentioned before, under thermodynamically controlled conditions, 3-oxocyclohexaneethanals of type **43** give by intramolecular aldol reaction an about 85/15 *endo/exo* 6-hydroxybicyclo[2.2.2]octan-2-one **44** mixture (Scheme 5). It was shown by our group that this equilibrium distribution is due to an unfavourable 1,3 boat-axial interaction experimented in the *exo* epimer by the *pseudo*-axially oriented hydroxy group [74]. It follows that, in a substituted system, the location of the carbonyl influences the orientation of the hydroxyl group.

**Scheme 6:**
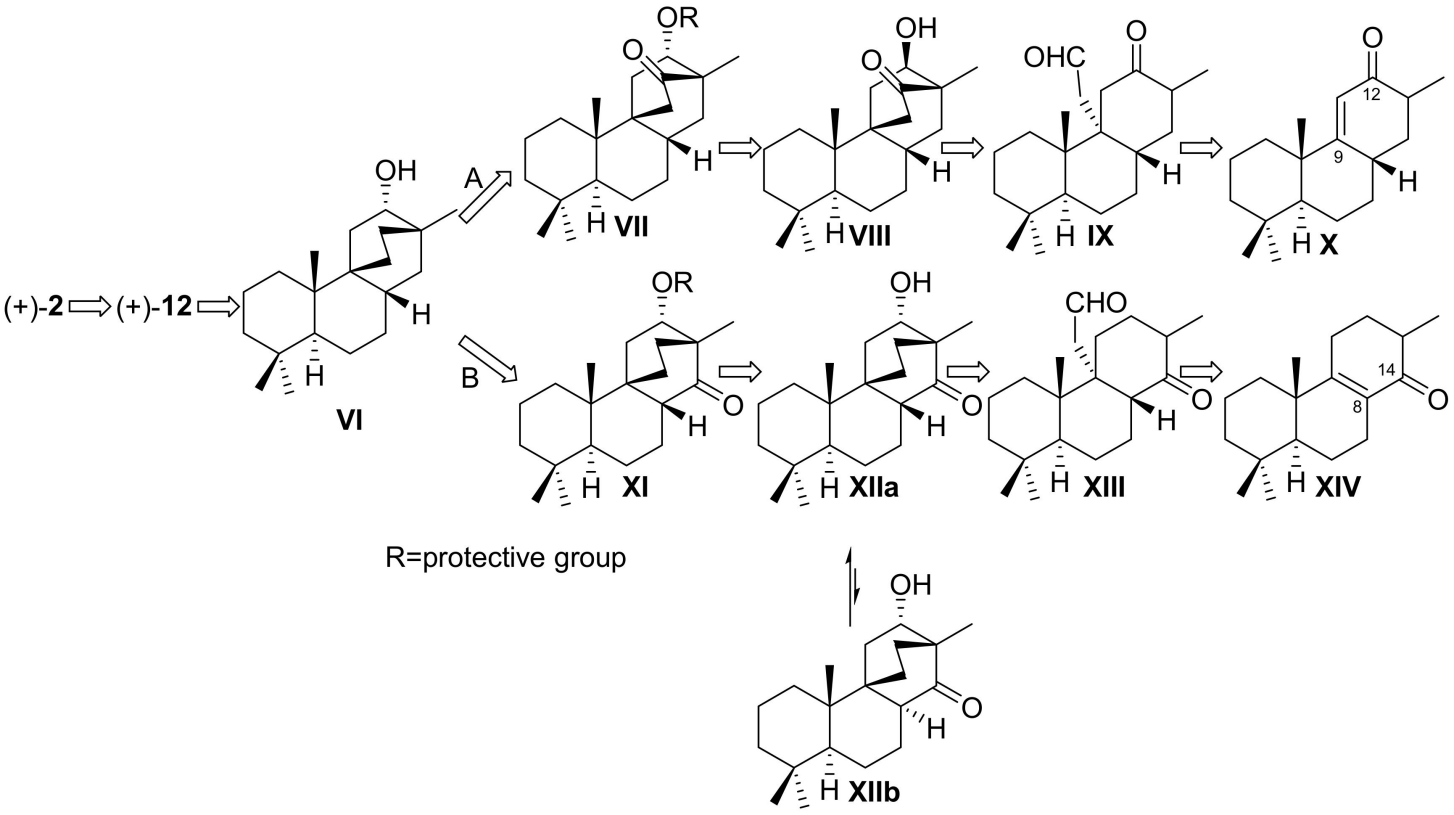
Retrosynthetic approaches to key intermediates of type **VI**.

We decided to solve the problem by the Wiesner two carbons annellation methodology. Two approaches were suggested by retrosynthetic analysis (Scheme 6): the first one (A), starting from a 9(11)-podocarpen-12-one, as in the Kelly approach, would have required the HO-C(12) configuration inversion; the second one (B) would have requested the photoaddition to be performed on a 8(9)-podocarpen-14-one, which, on the basis of the Wiesner empirical rule [57,58,59], should have ensured the same stereofacial selectivity and produced a 6-hydroxy-bicyclo[2.2.2]octan-2-one epimeric mixture whose major epimer should have the HO-(C12) group properly oriented (α) for the rearrangement to the stemarane system.

**Figure 7:**
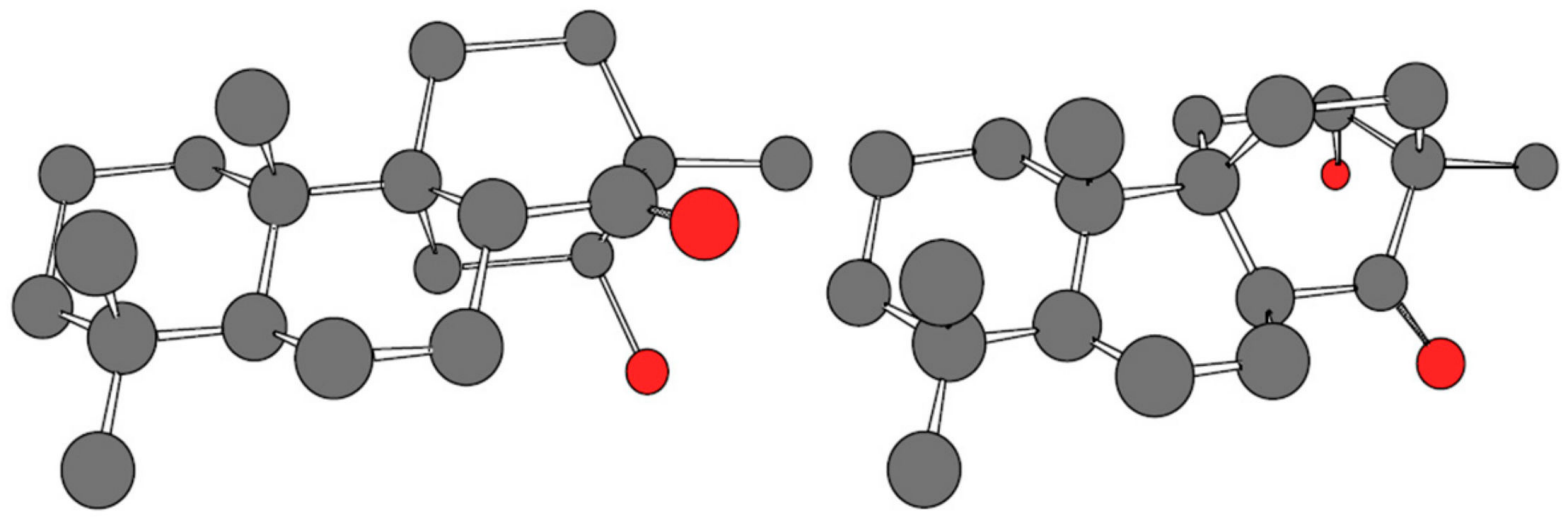
Three-dimesional structures of **XIIa** (left) and **XIIb** (right); red round indicates oxygen atom and grey one carbon atom.

In this approach, in the hydroxybiciclo[2.2.2]octanone intermediates **XII** the H-C(8) is adjacent to the C(14) carbonyl group. In principle, two epimers (**XIIa** and **XIIb**) at C(8) could be formed as result of the experimental conditions necessary for the intramolecular aldol reaction. Comparing the structures of **XIIa** and **XIIb** the epimer having the H–C(8) α configurated (**XIIb**) appears less stable because of the presence of a number of unfavourable steric interactions: while ring A is in a chair conformation ring B is in a boat conformation and the C(9)-C(10) and CH_3_-C(10) bonds are eclipsed (Figure 7, right). On the contrary in **XIIa** in which the H-C(8) is β configurated both rings A and B are in the chair conformation and the C(9)-C(10) and the CH_3_-C(10) bonds are staggered (Figure 7, left). After equilibration the C(8) stereogenic center should therefore materialize in the desired configuration in which the H-C(8) is β oriented.

#### 7.2.1. Approach A: Diastereoselective Synthesis of (+)-13-Stemarene 12 and (+)-18-Deoxystemarin 2 by Allene Addition to 9(11)-Podocarpen-12-one

**Scheme 7:**
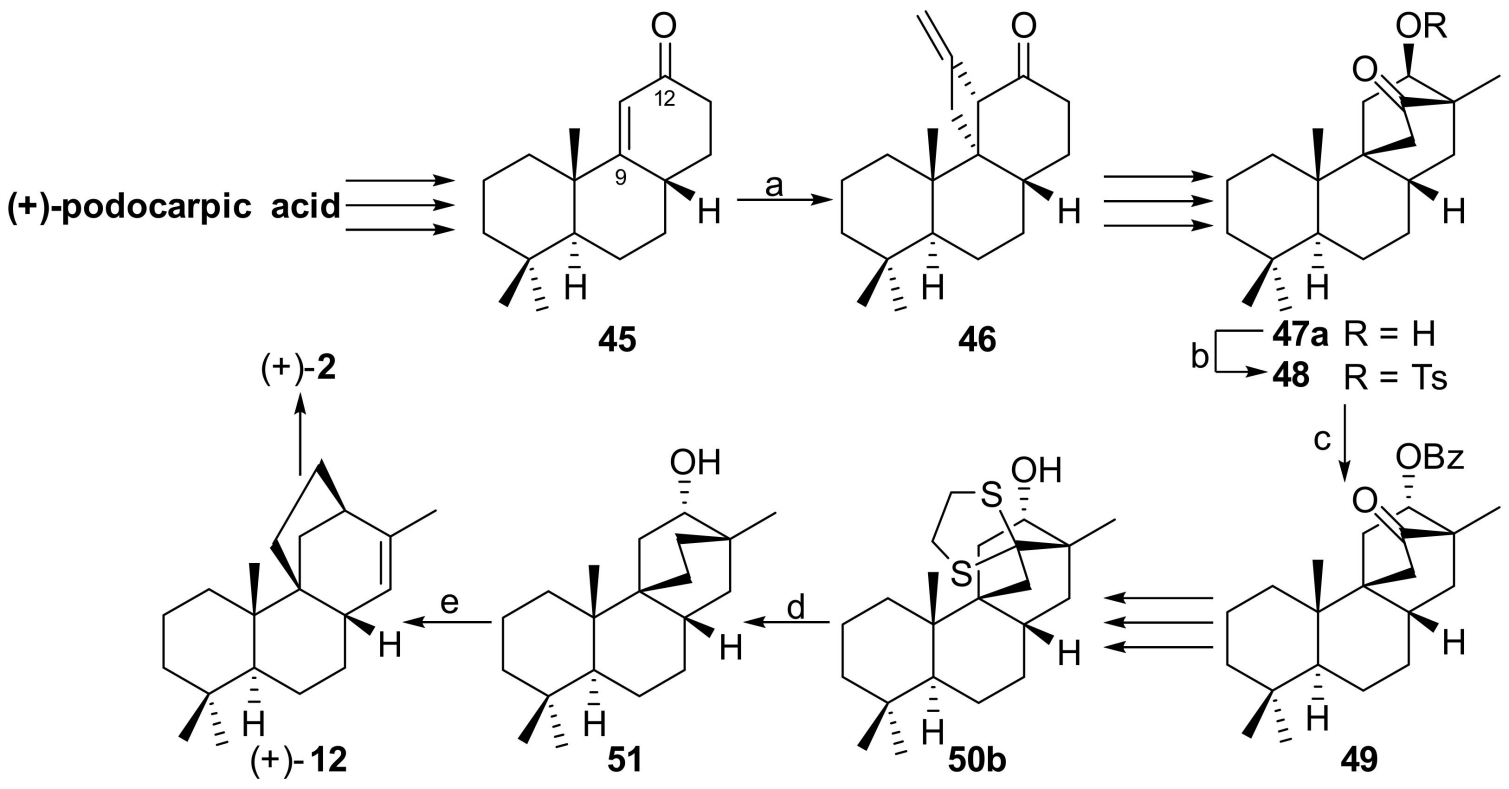
Diastereoselective synthesis of (+)-**12** and (+)-**2** fom (+)-podocarpic acid. Reagents and conditions: (**a**) allene, THF, hν, −78 °C; (**b**) TsCl/Py; (**c**) NEt_4_(PhCOO); (**d**) *Raney*-Ni, EtOH, reflux; and (**e**) TsOH, benzene, reflux.

This approach—which has been recently reviewed [75]—has been successfully accomplished and resulted in the synthesis from (+)-podocarpic acid of (+)-13-stemarene **12** and (+)-18-deoxystemarin **2** (Scheme 7) [14,76,77]. It was based on the inversion of configuration of the HO-C(12). Thus the major ketol epimer **47a** was converted into the corresponding tosylate **48** and the latter treated with Et_4_N(PhCOO) in acetone at reflux affording the *exo*-benzoate **49**. This methodology, described in the past by Streitweiser and co-workers for the inversion of configuration of acyclic secondary alcohols [78], is quite convenient in that produces a locked *exo*-ketol which cannot therefore re-equilibrate to the more stable *endo* epimer.

**Scheme 8:**
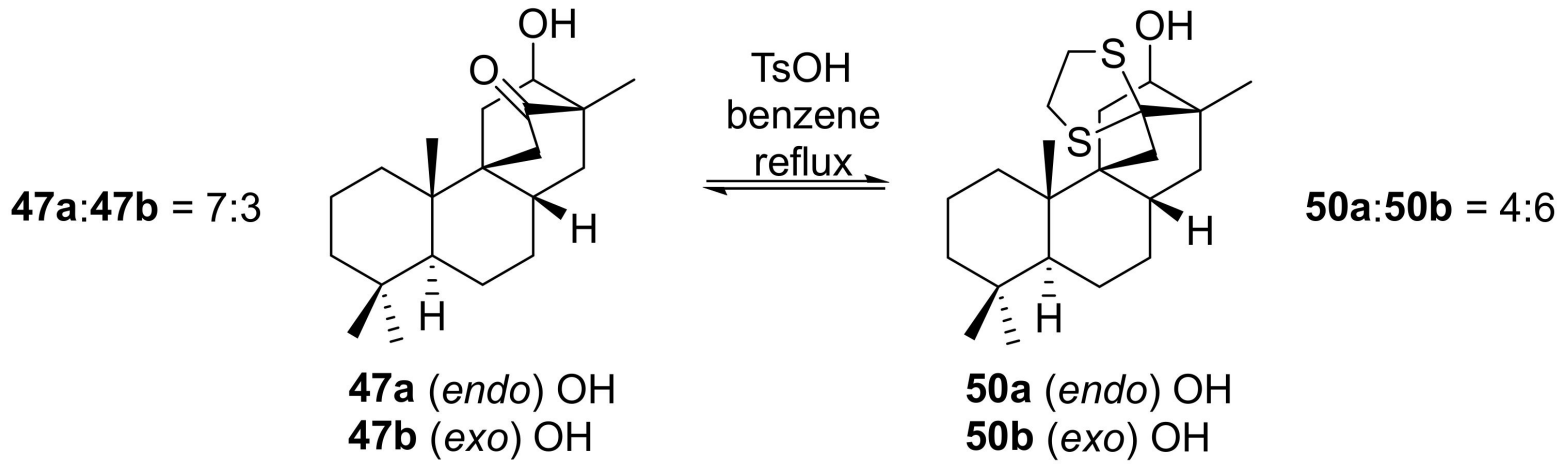
Ethylene dithioacetal equilibration.

As can be observed in Scheme 8, the 6-*exo*-hydroxybicyclo[2.2.2]octan-2-one ethylene dithioacetal **50b** is a key intermediate in the synthesis of 13-stemarene **12** and 18-deoxystemarin **2**.

Thus an expeditious preparation of **50b** was also elaborated by equilibrating under acidic conditions the *endo* rich 6-hydroxybicyclo[2.2.2]octan-2-one ethylene dithioacetal mixture **50**. It was found that, after equilibration, the *exo* epimer **50b** is the major one [79].

#### 7.2.2. Approach B: Attempted Diastereoselective Synthesis of (±)-Stemarin 1 by Allene Addition to a 8(9)-Podocarpen-14-one

The approach to stemarane diterpenes via a 8(9)-podocarpen-14-one appeared quite attractive since the allene photoaddition was expected, on the basis of the Wiesner empirical rule (*vide supra*), to proceed from the α-side, as in the case of 9(11)-podocarpen-12-ones, thus ensuring the correct stereochemistry at C(9). Besides, the HO-C(12) and H-C(8) should have both emerged from the aldol reaction in the desired orientation (*vide supra*).

**Scheme 9:**
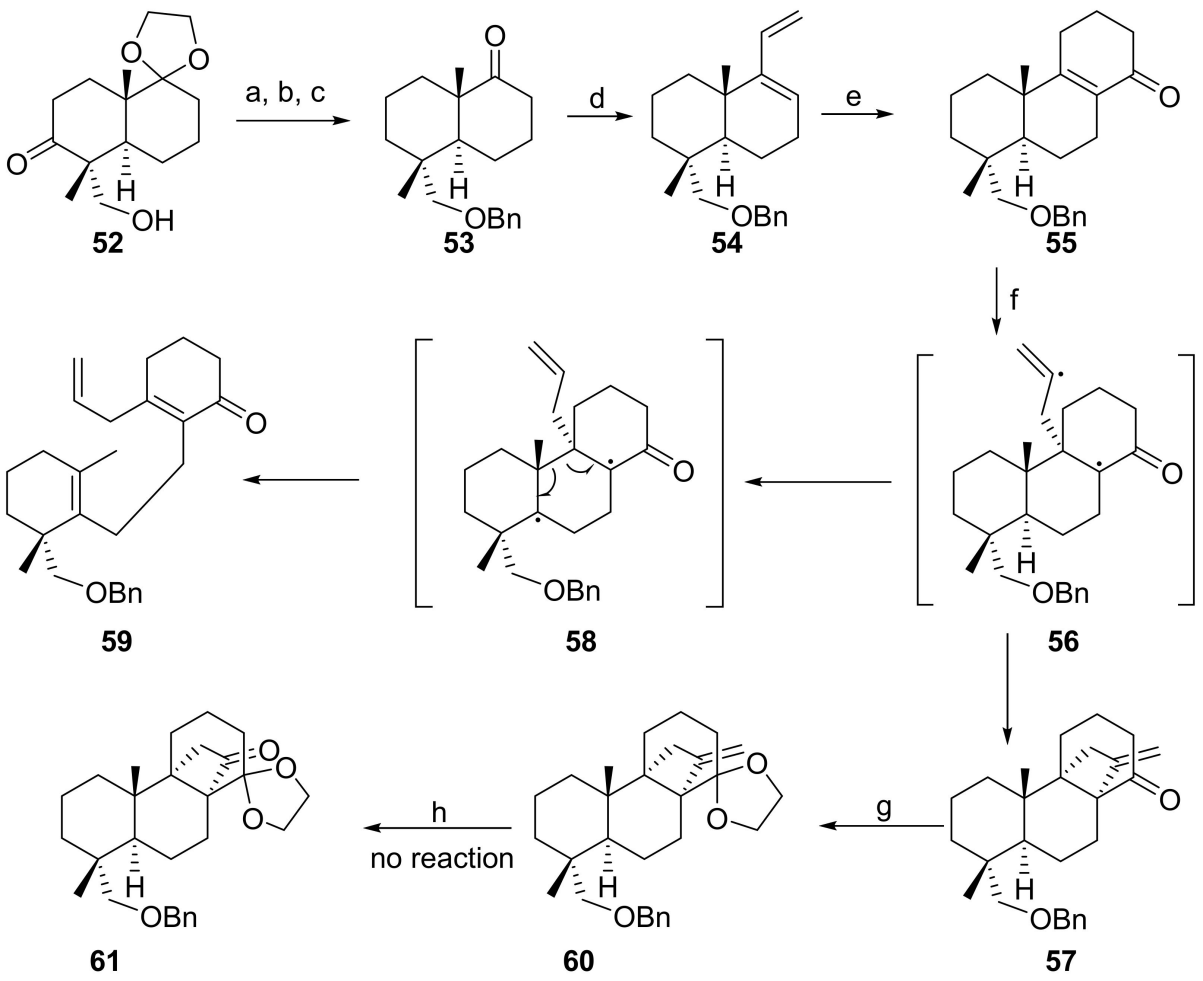
Attempted approach *via* 8(9)-podocarpen-14-one **46**. Reagents and conditions: (**a**) (1) TsNHNH_2_, THF, reflux, 3 h; (2) NaBH_3_CN, reflux, 8 h (**b**) (1) NaH, THF, reflux, 1 h; (2) BnBr, reflux, 3 h (**c**) 2N HCl, reflux, 30 min (**d**) (1) BrMgCHCH_2_, THF, 0 °C, N_2_; (2) PBr_3_, CH_2_Cl_2_, dimethylformamide, −10 °C, N_2_; 3 h 0 °C.; (**e**) α-acetoxycrilonitrile, Carius tube, 110 °C, N_2_, 3 d; (**f**) allene, hν, pyrex, −78 °C; (**g**) ethylene glycol, TsOH, benzene, Dean–Stark; and (**h**) OsO_4_/NaIO_4_.

Surprisingly, after a successful preparation of the 8(9)podocarpen-14-one intermediate **46** [80] and a model work [81], the allene photoaddition to 8(9)-podocarpen-14-one **55**, obtained from known **52** [82,83], gave, along the expected cyclo-photoadduct **57**, compound **59** [84]. Compound **59** is the result of the H-C(5) abstraction by the initially side chain radical **56**, to give the Cope rearrangement like intermediate **58**, followed by homolytic cleavage of the C(9)-C(10) bond (Scheme 9). Photoadduct **57** was then acetalized with ethylene glycol to give **60**. Unfortunately, we were unable to cleave the exocyclic double bond into **61** (Unpublished results by our group.). This approach was therefore discontinued.

### 7.3. Regio- and Diastereoselective Synthesis of (+)-Stemar-13-ene 12 and (+)-18-Deoxystemarin 2 by the 6-Hydroxy-1-methylbicyclo[2.2.2]octan-2-one → 4-Methylbicyclo[3.2.1]oct-3-en-6-one Skeletal Rearrangement

**Scheme 10:**
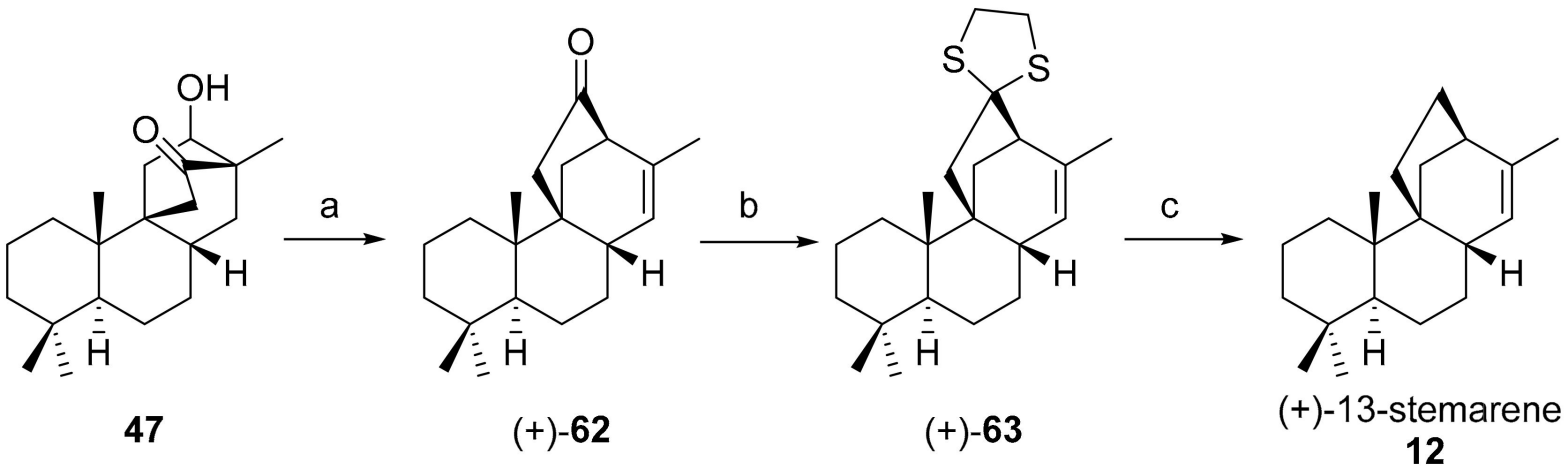
Reagents and conditions: (**a**) TsOH, toluene, reflux; (**b**) 1,2-ethanedithiol, BF_3_·Et_2_O, 0 °C, 85%; and (**c**) *Raney*-Ni, EtOH, 60 °C, 76%.

This approach, which has been recently reviewed, was successfully accomplished by our group resulting in the synthesis of (+)-13-stemarene **12** and (+)-18-deoxystemarin **2**. It was based on the 6-hydroxy-1-methyl-bicyclo-[2.2.2]octan-2-one → 4-methylbicyclo[3.2.1]oct-3-en-6-one skeletal rearrangement described in 2006 by Srikrishna and co-workers at the Indian Institute of Science, Bangalore, in the frame of a study on the reactivity of isotwistanes [85]. The remarkable feature of this approach [86] is that, owing to the stereospecificity of the rearrangement and to the 1-methyl-6-hydroxybicyclo[2.2.2]octan-2-ones *endo/exo* equilibrium, the whole 1-methyl-6-hydroxybicyclo[2.2.2]octan-2-ones *endo/exo* mixture **47** is converted into the rearrangement product in which is also present the C(13)-C(14) double bond (Scheme 10), a characteristic feature of some stemarane diterpenoids such as (+)-oryzalexin S **11** and a necessary tool for the introduction of the α configurated HO-C(13) if necessary.

### 7.4. Regio- and Diastereoselective Synthesis of (+)-2-Deoxyoryzalexin S 2 from (+)-Podocarpic Acid

**Scheme 11:**
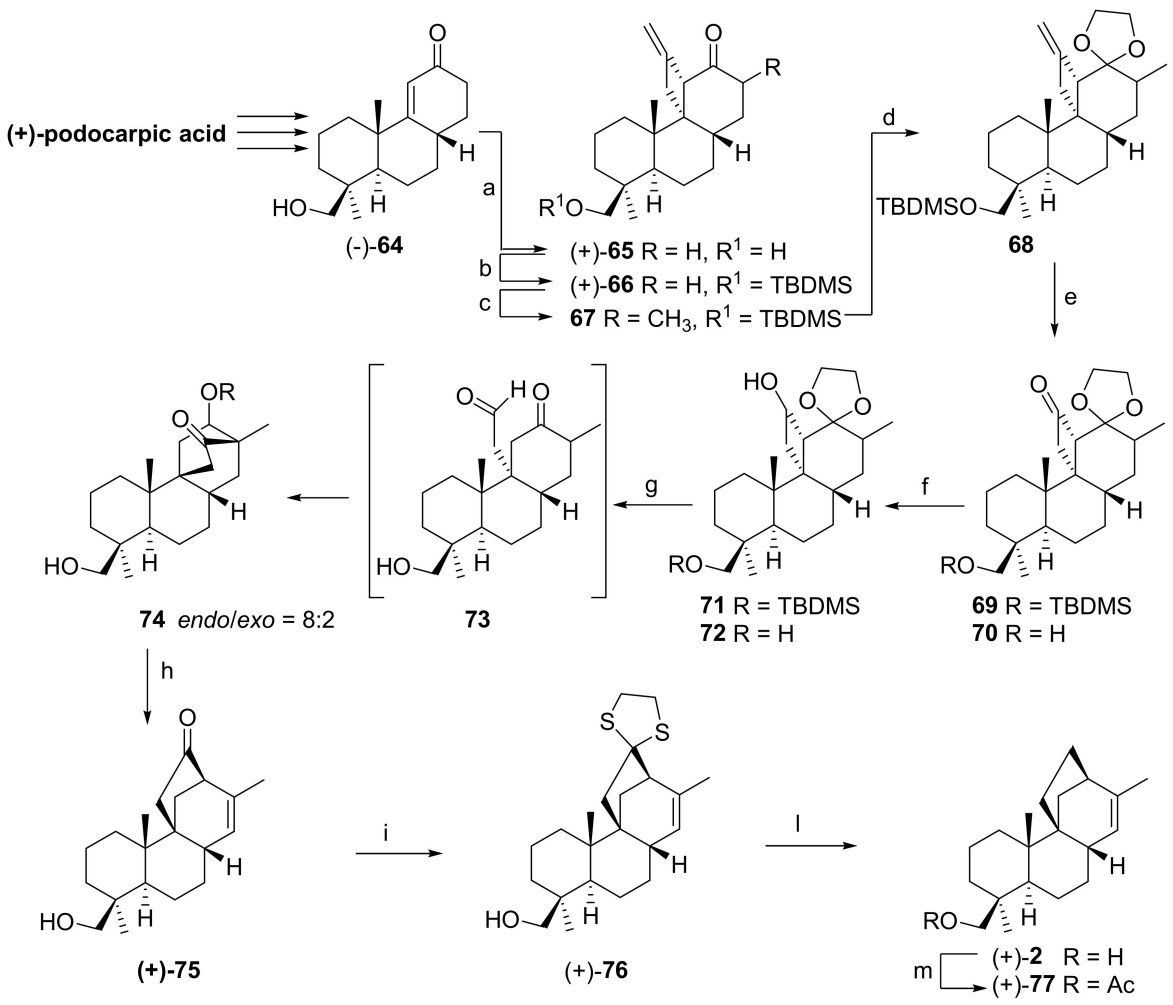
Reagents and conditions: (**a**) allene, THF, −78 °C, hν, 81%; (**b**) TBDMSCl, imidazole, THF, 89%; (**c**) NaHDMS, CH_3_I, THF, −78 °C; (**d**) ethylene glycol, toluene, TsOH 78%; (**e**) OsO_4_/NaIO_4_, THF, H_2_O; (**f**) NaBH_4_, EtOH/MeOH, 0 °C; (**g**) THF/2N HCl, 88%; (**h**) TsOH, benzene, 85 °C, 66%; (**i**) 1,2-ethanedithiol, BF_3_·Et_2_O, 0 °C, 84%; (**l**) Raney-Ni, EtOH, 60 °C, 62%; and (**m**) Ac_2_O/Py, 99%.

Finally, having in hand an efficient methodology for the construction of the C/D ring system of stemarane diterpenoids, we decided to apply it to the synthesis of (+)-2-deoxyoryzalexin S **2** the structure and absolute configuration of which had been established only on the basis of the ^1^H and ^13^C spectra [2,3,5]. The strategy adopted is described in Scheme 11. The starting material was (−)-19-hydroxypodocarp-9-en-12-one **64**, available in four steps from (+)-podocarpic acid. Photoaddition of allene to (−)-**64** in THF at 78 °C gave quantitatively the photoadduct (+)-**65** the structure of which was established by 2D NMR experiments. Prior to methylation at C(13), the HO-C(19) in (+)-**65** was protected to give (+)-**66**. Compound (+)-**66** was then methylated to give **67**. The latter was then converted into the acetal **68**, and the exocyclic methylene cleaved with OsO_4_/NaIO_4_ to give the cyclobutanone **69** along with some unprotected keto-alcohol **70**. Compound **69** was then reduced to the cyclobutanol **71**. Treatment of **71** with a 2:1 THF/2N HCl mixture gave **74** as an about 80:20 *endo/exo* C(12) epimeric mixture. The latter dissolved in toluene was heated at 85 °C in the presence of TsOH, giving (+)-**75**. Thioacetalization of (+)-**75** by standard methods followed by deoxygenation gave then (+)-2-deoxyoryzalexin S **2** which was then acetylated to (+)-**77** [12]. Remarkably, in this work all chiral centers present in (+)-podocarpic acid are maintained.

The relative configuration of (+)-**77** was confirmed by an X-Ray structure determination. This work allowed us to demonstrate that the structure of (+)-2-deoxyoryzalexin S **2** could not be attributed to a Chilean *Calceolaria* isolated diterpenoid to which this structure had been assigned.

### 7.5. Other Strategies

**Scheme 12:**
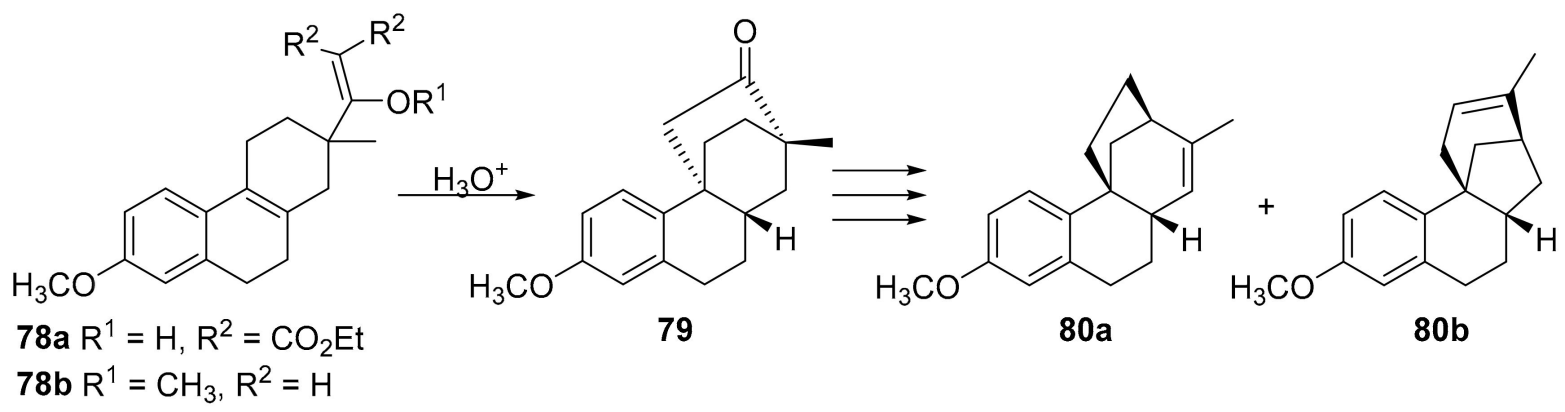
Approach by Ghatak and co-workers to stemarane and stemodane diterpenoids.

An approach to stemodane and stemarane diterpenoids was also described in 1985 by Ghatak and co-workers at the Indian Association for the Cultivation of Science, Jadavpur, Calcutta [87]. This approach (Scheme 12) was based on the formation of a bicyclo[2.2.2]octane intermediate by acid-catalyzed intramolecular C-alkylation followed by a [2.2.2] → [3.2.1] rearrangement (*vide supra*).

**Scheme 13:**
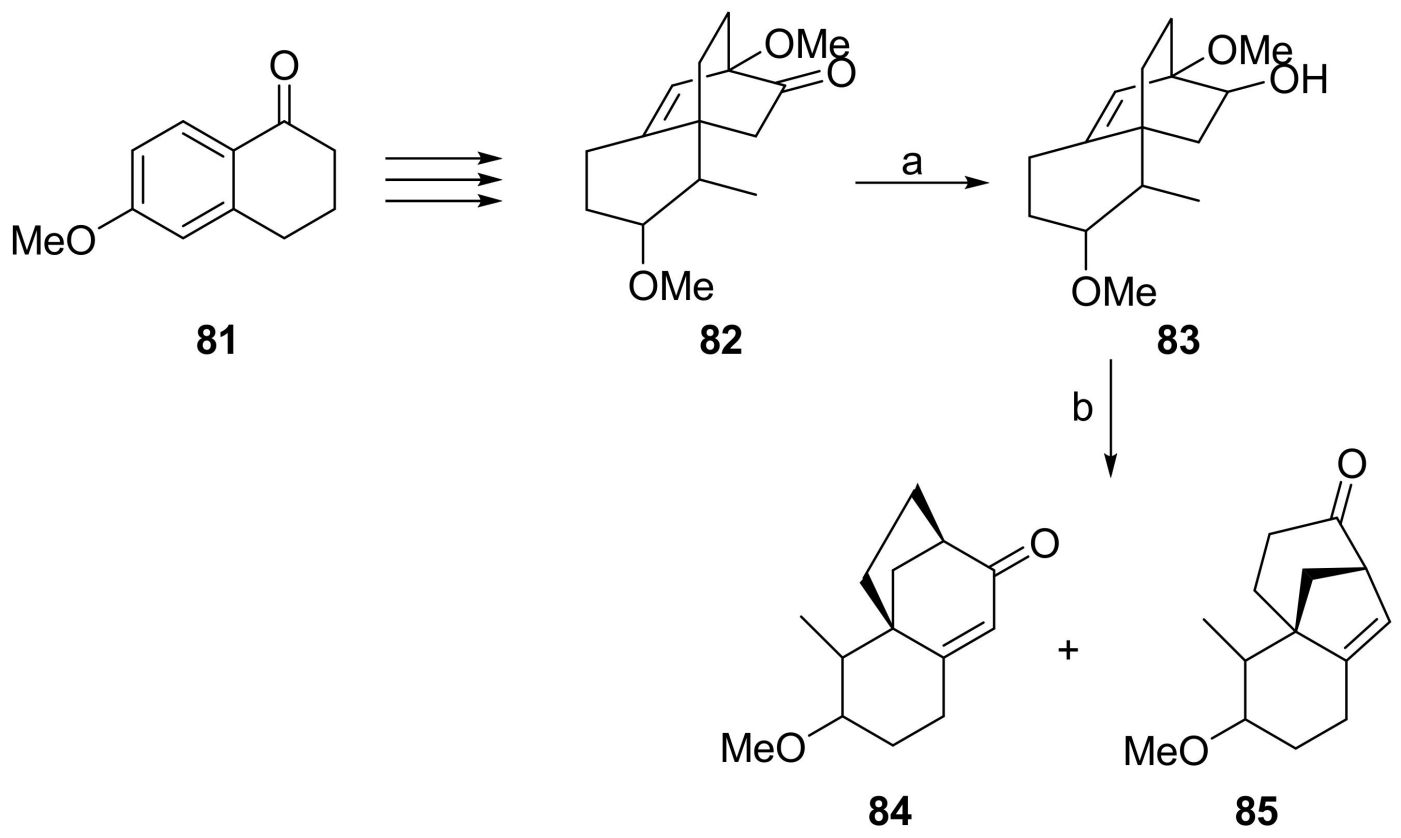
Subba Rao’s and Kaliappan’s approach to stemarane and aphidicolane diterpenoids: reagents and conditions: (**a**) NaBH_4_, EtOH; and (**b**) BF_3_.Et_2_O or MsCl/Et_3_N.

A short, expedient, though not diastereoselective route, *inter alia*, to a potentially key intermediate for their synthesis of stemarane diterpenoids was also realized by Subba Rao and Kaliappan at the Indian Institute of Science, Bangalore, India (Scheme 13) [88]. Also this approach, to our best knowledge, was not implemented with a total synthesis.

### 7.6. Enantioselective Synthesis of Stemarane Diterpenes and Diterpenoids via 9(11)-Podocarpen-12-ones

**Scheme 14:**
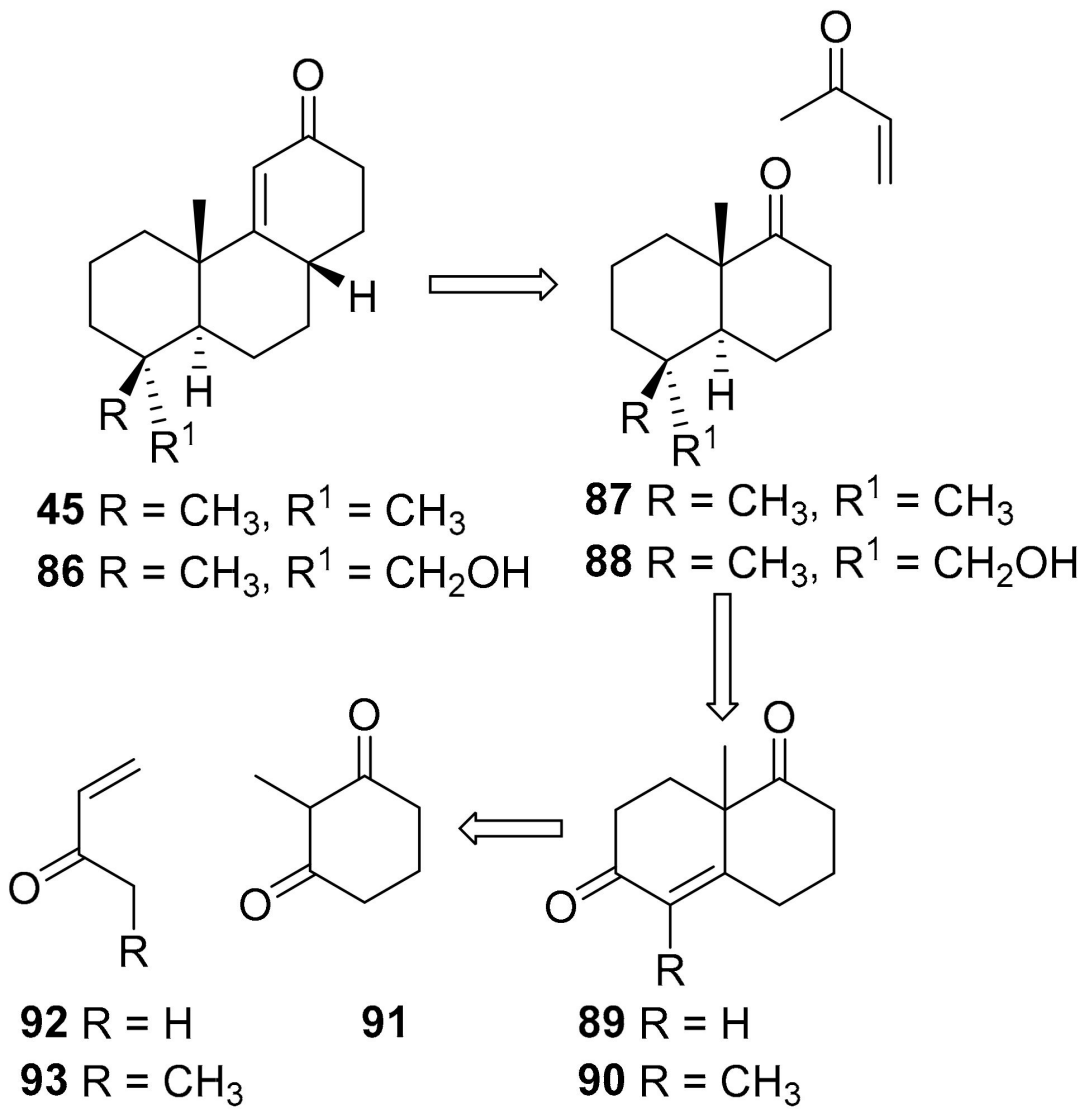
Retrosynthetic analysis for the obtaining of substituted 9(11)podocarpen-12-ones.

9(11)-podocarpen-12-ones such as **45** can be obtained, *inter alia*, by Robinson annulation of a substituted decalone with methyl vinyl ketone (Scheme 14). At the end of the annulation process, the vinilogous H-C(8) results installed in the more stable β configuration [70,72,89].

In turn the decalone intermediate such as **87** can be obtained from the Wieland–Miescher ketone. Since Wieland–Miescher ketone **89** and its C(4) homologue **90** can be obtained enantioselectively [90,91,92,93,94], this approach is quite convenient for the preparation of optically active 9(11)podocarpen-12-ones and hence of the target compounds.

Thus decalone **87** [95] was used to prepare (+)-9(11)podocarpen-12-one **45** and hence (+)-13-stemarene **12** and (+)-2-deoxystemarin **2**. Decalone **88** [95] could be adopted in principle for the obtaining of (+)-stemarin **1**.

**Scheme 15:**
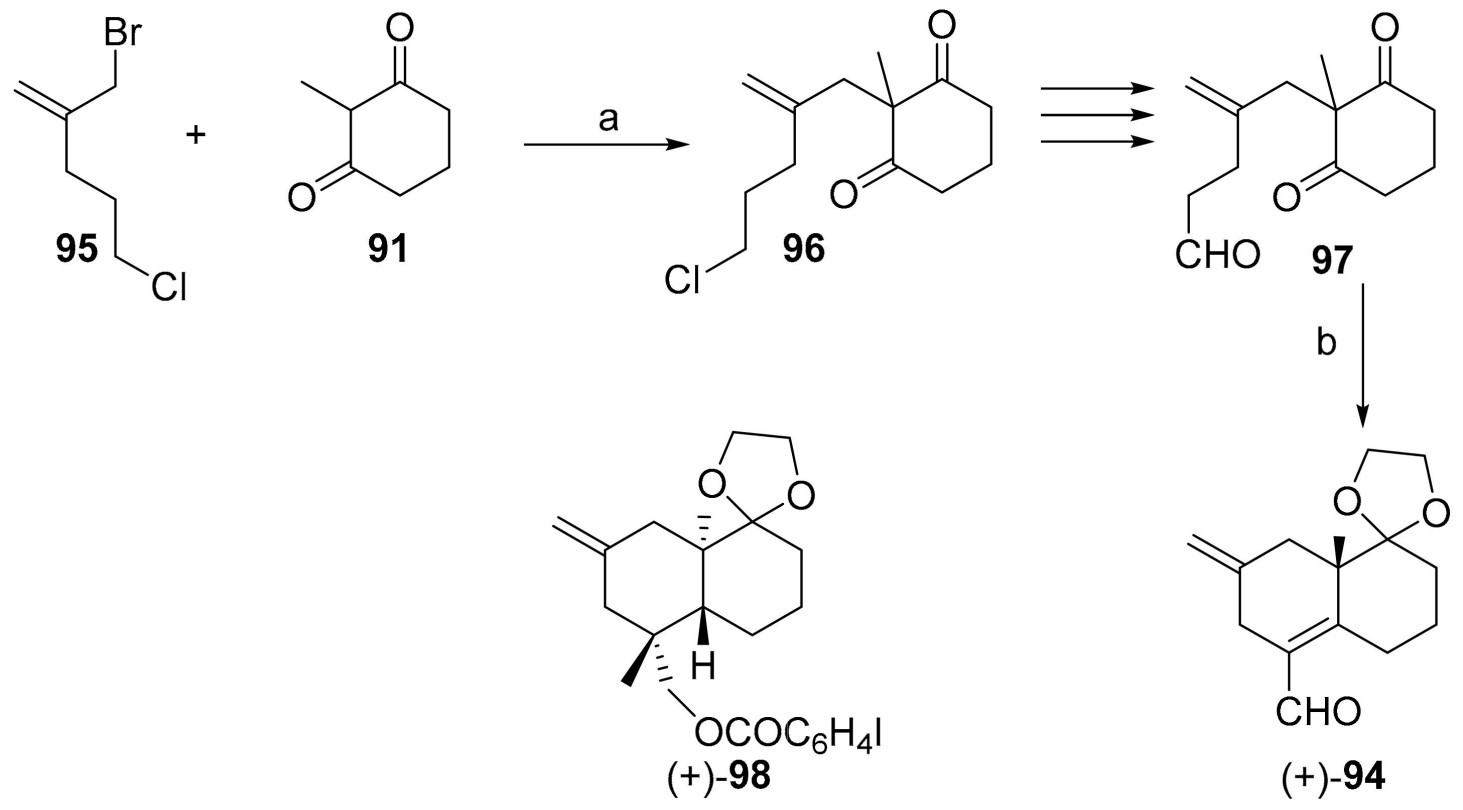
Enantioselective obtaining of (+)-**94**. Reagents and conditions: (**a**) (1) NaH/DMF, 1 h, Ar; (2) then **95**, 2 h; and (**b**) DMSO/1 M HClO_4_, d-tyrosine, 10 °C, 20 min.

Recently, the synthesis of the bicyclic intermediate **94**, necessary for the enantioselective obtaining of (+)-oryzalexin S **11** was described by our group (Scheme 15) [96].

To this end, it was necessary to elaborate the *ad hoc* side chain **95** to be used in the annulation process leading to ring A. The exocyclic double bond at C(2) would have easied the alkylation of 2-methyl-1,3-cyclohexanedione **91** and allowed its conversion at proper time into the α configurated HO-C(2).

The halide at the end of the side chain would have allowed the introduction of the formyl group necessary for the cyclization. Later the latter group could be converted in the HO-C(18).

Carrying out the aldol reaction in the presence of d-tyrosine at 10 °C the enantioselective cyclization of **97** to **94** was accomplished in very good yield and e.e. The obtaining of **94** followed an X-ray structure determination on **98**. (Initially the work was carried out with the cheapest l-amino acid series.)

## 8. Conclusions

In this paper, the work by various research groups on stemarane diterpenes and diterpenoids, described in over forthy papers covering isolation, structure elucidation, biogenesis, biosynthesis, biotranformations, synthesis, and enantiosynthesis has been reviewed. From this review, it appears that further work is necessary to establish unambiguously the structure and absolute configuration of stemarane diterpenes and diterpenoids from the Chilean flora. In fact, while the structure and absolute configuration of (+)-stemarin **1** was established by X-ray diffraction, the structure and absolute configuration of stemarane diterpenes and diterpenoids from the Chilean flora was not confirmed by chemical correlation nor by a X-ray structure determination. The biogenesis proposed for this class of compounds was confirmed by several biosynthetic studies. Biotransformations under the activity of a number of fungi leading to interesting metabolites were also carried out. Besides, it appears that the biological activity of pure isolated stemarane diterpenes and diterpenoids, but (+)-oryzalexin S **11**, has not been evaluated yet. The biological activity ascertained so far was attributed to *Stemodia maritima* L. metabolites, which differ from stemarane diterpenes and diterpenoids. Finally, a comparison about the content of stemarane diterpenes and diterpenoids among *Stemodia maritima* L. plants, collected in different geographical areas, seems also an interesting task. The extensive work towards the synthesis of this class of compounds resulted in a very efficient approach while recent studies allowed the enantiosynthesis of a key intermediate for the synthesis of (+)-oryzalexin S **11**, and should pave the way to this interesting and bioactive compound. We hope this review will be useful to those who are interested in this field.
